# Different Types of Diatom-Derived Extracellular Polymeric Substances Drive Changes in Heterotrophic Bacterial Communities from Intertidal Sediments

**DOI:** 10.3389/fmicb.2017.00245

**Published:** 2017-02-27

**Authors:** Julio Bohórquez, Terry J. McGenity, Sokratis Papaspyrou, Emilio García-Robledo, Alfonso Corzo, Graham J. C. Underwood

**Affiliations:** ^1^Department of Biology, Faculty of Marine and Environmental Science, University of CádizPuerto Real, Spain; ^2^School of Biological Sciences, University of EssexColchester, UK; ^3^Departamento de Biomedicina, Biotecnología y Salud Publica, Universidad de CádizPuerto Real, Spain; ^4^Microbiology Section, Department of Biosciences, University of AarhusAarhus, Denmark

**Keywords:** EPS, microphytobenthos, intertidal sediment, nutrient regeneration, G-model, degradation rate, microbial community, pyrosequencing

## Abstract

Intertidal areas support extensive diatom-rich biofilms. Such microphytobenthic (MPB) diatoms exude large quantities of extracellular polymeric substances (EPS) comprising polysaccharides, glycoproteins and other biopolymers, which represent a substantial carbon pool. However, degradation rates of different EPS components, and how they shape heterotrophic communities in sediments, are not well understood. An aerobic mudflat-sediment slurry experiment was performed in the dark with two different EPS carbon sources from a diatom-dominated biofilm: colloidal EPS (cEPS) and the more complex hot-bicarbonate-extracted EPS. Degradation rate constants determined over 9 days for three sediment fractions [dissolved organic carbon (DOC), total carbohydrates (TCHO), and (cEPS)] were generally higher in the colloidal-EPS slurries (0.105–0.123 d^−1^) compared with the hot-bicarbonate-extracted-EPS slurries (0.060–0.096 d^−1^). Addition of hot-bicarbonate-EPS resulted in large increases in dissolved nitrogen and phosphorous by the end of the experiment, indicating that the more complex EPS is an important source of regenerated inorganic nutrients. Microbial biomass increased ~4–6-fold over 9 days, and pyrosequencing of bacterial 16S rRNA genes revealed that the addition of both types of EPS greatly altered the bacterial community composition (from 0 to 9 days) compared to a control with no added EPS. Bacteroidetes (especially *Tenacibaculum*) and Verrucomicrobia increased significantly in relative abundance in both the hot-bicarbonate-EPS and colloidal-EPS treatments. These differential effects of EPS fractions on carbon-loss rates, nutrient regeneration and microbial community assembly improve our understanding of coastal-sediment carbon cycling and demonstrate the importance of diverse microbiota in processing this abundant pool of organic carbon.

## Introduction

Microphytobenthic communities inhabiting intertidal sediments, such as salt marshes and mudflats, exhibit high rates of primary production (Underwood et al., 2005) and are able to influence carbon and nitrogen fluxes in shallow-water systems (Perkins et al., 2001; Thornton et al., 2002; McKew et al., 2013). Benthic diatoms are the major autotrophic microphytobenthic group in fine (cohesive) intertidal sediments, and can contribute up to 50% of the autochthonous carbon fixation in some ecosystems (Underwood et al., 2005). This productivity contributes to the ecosystem service and carbon and nitrogen cycling provision of coastal habitats (Beaumont et al., 2014; Luisetti et al., 2014). Benthic diatoms inhabit the first few millimeters of the sediment and exude extracellular polymeric substances (EPS), which play important ecological roles including motility of the pennate diatoms (Underwood and Paterson, 2003; Hanlon et al., 2006) and protection of cells from desiccation and salinity stress (Steele et al., 2014). In addition, EPS are used by bacteria, meio- and macrofauna as carbon and energy sources (Middelburg et al., 2000; Haynes et al., 2007; Bellinger et al., 2009) and contribute to sediment stability (Underwood and Paterson, 2003; Ubertini et al., 2015).

Diatom EPS include a wide range of different organic macromolecules, primarily polysaccharides (up to 90%: Underwood et al., 2010), but also glycoproteins and lesser amounts of lipids, nucleic acids and proteins (Hoagland et al., 1993; Underwood and Paterson, 2003; Hofmann et al., 2009). EPS is operationally described as material that precipitates in polar solvents (Decho, 1990), and can be separated by extraction procedures, for example. colloidal EPS (EPS_coll_), water-soluble polymeric material isolated from colloidal aqueous extracts, and hot-bicarbonate extracted EPS (EPS_HB_), higher molecular weight (HMW) and more insoluble compounds such as tightly bound and capsular EPS, solubilized using hot bicarbonate extraction protocols (Bellinger et al., 2005; Aslam et al., 2016). These labile and bound EPS fractions differ in biochemical composition, and in the seasonal changes of their chemical composition (Pierre et al., 2014; Passarelli et al., 2015).

Carbohydrate content varies between 40 and 90% of the EPS-carbon within diatom-rich biofilms (Underwood and Smith, 1998; Underwood and Paterson, 2003). The response of the heterotrophic bacterial community to this carbon source depends on its biochemistry, with bacteria having to deploy extracellular enzymes to transform the more complex EPS molecules into smaller monomers and oligosaccharides prior to uptake (Hofmann et al., 2009; Thornton et al., 2010; Arnosti, 2011). Previous slurry experiments have shown rapid utilization, within hours, of low-molecular-weight compounds, followed by a slower rate of degradation of more complex EPS by particular groups of bacteria (Haynes et al., 2007; Hofmann et al., 2009). The diversity of EPS composition results in a great variety of bacteria being involved in their degradation (Bacteroidetes, together with Alpha-, Beta- and Gammaproteobacteria, including *Acinetobacter*) (Elifantz et al., 2005; Haynes et al., 2007; McKew et al., 2013; Taylor et al., 2013; Passarelli et al., 2015). Coupling between diatom-derived EPS and bacterial community composition has been demonstrated (Taylor et al., 2013; Miyatake et al., 2014); nevertheless, still little is known about the loss processes affecting EPS budgets in intertidal sediments, and in particular on the differential degradation of the range of EPS produced within biofilms (McKew et al., 2013).

In order to better understand the effect of EPS composition on its turnover, we performed a microcosm experiment using slurries from intertidal diatom-dominated sediment enriched with two different carbon sources: colloidal EPS (EPS_coll_), and more tightly-bound, extracellular components of the capsular EPS associated with the surface of the diatom frustules (EPS_HB_), extracted from natural MPB biofilms. Our hypothesis was that due to the differences in structural complexity, the degradation rate of EPS_coll_ would be faster than for EPS_HB_, and that the dominant groups of bacteria would change in relation to their preference and ability to respond to the different EPS components. Since EPS, in addition to carbohydrates, contain a certain amount of proteins, glycoproteins and phospholipids (Hoagland et al., 1993; Underwood and Paterson, 2003; Hofmann et al., 2009), we hypothesized that EPS degradation might be a (previously unknown) source of regenerated nutrients (N and P) in marine sediments.

In order to test these hypotheses, we compared changes in EPS-enriched slurries of: (1) concentrations of Dissolved Organic Carbon (DOC), which contains mainly carbohydrate and also amino acids and other low-molecular-weight organic carbon compounds; (2) concentrations of Total Carbohydrates (TCHO), which includes all dissolved and particulate carbohydrate and also structural polysaccharides; (3) concentrations of cEPS, (4) changes in the concentrations of inorganic nutrients, and (5) biomass and bacterial community assemblages based on DNA assays.

## Materials and methods

### Sampling site and extraction of carbohydrates fractions

Surface sediment (top 2 mm depth) was collected in October 2012 from a tidal mudflat near Alresford creek (Colne Estuary) (51°50′14.9″N, 0°59′35.2″E) (UK), where abundant diatom-dominated biofilms were present. Sediment was frozen at −20°C for 12 h and freeze-dried overnight. Two different operational EPS fractions (Underwood and Paterson, 2003) were extracted (multiple extractions of 5 g of sediment) from the freeze-dried sediments, following a sequential extraction procedure. First, the colloidal EPS fraction (EPS_coll_) of water-soluble carbohydrate fractions was obtained (Decho, 1990). Then, after a hot-water extraction (to remove intracellular carbohydrates), a hot-bicarbonate (HB) solubilization step was performed (addition of 0.5 M NaHCO_3_ solution at 95°C for 1 h) to obtain a fraction containing gelatinous extracellular polysaccharides termed EPS_HB_ (Bellinger et al., 2005).

The supernatant containing either EPS_coll_ or EPS_HB_ was precipitated in ethanol (70% final concentration) overnight at 4°C, then centrifuged at room temperature (3,000 × g, 15 min), the supernatant discarded and the resultant EPS pellets from the parallel extractions pooled and resuspended in 400 mL of deionized water. The EPS_coll_ and EPS_HB_ extracts were dialyzed at room temperature overnight through an 8 kDa dialysis tubing against ultrapure water (18.2 MΩ cm, Milli-Q) with moderate stirring, to reach a final salinity <1%0. Subsamples of EPS_coll_ and EPS_HB_ extracts were measured spectrophotometrically (485 nm) after a phenol-sulfuric acid assay reaction (Dubois et al., 1956) as described by Hanlon et al. (2006). Carbohydrate concentration was quantified (μg mL^−1^) as glucose equivalents (later transformed to μmol C L^−1^) using a *D*-glucose standard curve. The final EPS_coll_ and EPS_HB_ extracts (215 mg C L^−1^) were kept in the dark at 4°C, and subsamples used for amendment of sediment slurry experiments.

### Experimental microcosms

Fresh sediment (top 2 mm) from the same location as that used for the EPS extraction was sampled on 3rd December 2012 and used to make the sediment slurries within 1 day. Five different slurry treatments were prepared each in triplicate 100 mL conical flasks: (1) +EPS_coll_ treatment consisted of 18.5 mL EPS_coll_ extract, 61.5 mL artificial sea water (salinity 35) and 200 mg wet weight estuarine sediment (containing ~120 μg DOC, McKew et al., 2013). As a result, the slurry had a total volume of 80 mL with a salinity of 27 and a dissolved carbon concentration of 51.5 mg C L^−1^; (2) the +EPS_HB_ treatment was established using an identical setup but with the addition of Hot-Bicarbonate-extracted EPS instead of EPS_coll_ extract having the same final carbon concentration; (3) NoSed-EPS_coll_ and (4) NoSed-EPS_HB_ controls had the same amount of the relevant EPS extract and artificial sea water as before but with no sediment added to check for abiotic loss; and (5) NoAdd-EPS control contained sediment and artificial sea water but no additional carbon source to check for changes in bacterial composition that are not a consequence of growing on the added EPS.

The flasks were placed on a rocking platform (100 r. p. m.) at 16°C in the dark to avoid an increase of carbon content as result of primary production. Samples were taken every 3 days for a total of 9 days.

### Organic carbon and carbohydrates

At each sampling time, subsamples of slurries (8 mL) were taken from each flask to measure organic carbon and carbohydrates in three inter-related fractions. Aliquots of 2.5 mL were filtered through pre-combusted GF/F filters, then diluted 10-fold with Milli-Q water to measure DOC on a Shimadzu TOC-VCSH Analyzer. An aliquot of slurry (4.5 mL) was centrifuged (3,000 × g, 15 min), after mixing by vortexing to remove the sediment particles and obtain a supernatant containing the colloidal material. The resultant supernatant was used to obtain the cEPS by precipitation in ethanol (70% final concentration). The remaining non-filtered 1 mL aliquot was used to measure TCHO. This fraction includes dissolved and colloidal carbohydrates as well as HW and HB fractions. Both cEPS and TCHO were quantified using the phenol-sulfuric acid assay as mentioned previously.

To quantitatively assess the degradability of the different organic fractions in the treatments, time course changes of the three carbon fractions, DOC, TCHO, and cEPS, were modeled in two ways. First, in order to facilitate comparison with other studies where only lineal degradation rates were provided, a linear degradation model according to Equation (1):

(1) $$\begin{array}{l} G_{t}=G_{o}-b\cdot t \end{array}$$

Secondly, the so-called one-G model of organic matter degradation (Berner, 1964) was implemented using the following exponential equation:

(2) $$\begin{array}{l} G_{t}=G_{o}^{-kt} \end{array}$$

where, *G*_*t*_ is the concentration of the organic fraction at time *t, G*_*o*_ is the initial concentration, *b* is the lineal degradation rate (μmol C_org_ L^−1^ d^−1^) and *k* is the degradation constant in d^−1^ units.

### Inorganic nutrients

A portion of slurry (8 ml for days 0 and 9, and 4 ml for days 3 and 6) was transferred to a 15 ml Falcon tube, centrifuged at 3,000 × g for 10 min, the supernatant was filtered using pre-combusted GF/F filters, and frozen immediately at −20°C, and later used to measure dissolved inorganic nutrients. Nitrate (${\text{NO}}_{3}^{-}$), nitrite (${\text{NO}}_{2}^{-}$) and ammonium (${\text{NH}}_{4}^{+}$), phosphate (${\text{PO}}_{4}^{3-}$) and silicate (${\text{SiO}}_{4}^{4-}$) were measured on a Seal Analytical AA3 HR Nutrient Autoanalyzer following the protocols described by Grasshoff et al. (1983).

To quantitatively assess the net rate of inorganic nutrient regeneration, time course changes in nutrients were fitted to a linear equation, where the slope represents the net regeneration rate. With some nutrients and in some treatments the use of a positive exponential model improved the correlation coefficients but we used a linear model in all cases to facilitate comparison.

### Bacterial community analysis

#### DNA extraction and microbial biomass estimation

The pellet obtained after centrifuging the slurry for nutrients analysis was retained and frozen at −20°C until DNA extraction. DNA was extracted from sediment pellets using a bead-beating phenol-chloroform-isoamyl alcohol method as described previously (McKew et al., 2011).

Small aliquots (1 μL) of extracted total DNA were diluted and used as a proxy to estimate the total biomass of the microbial community with a NanoDrop® 3,300 fluorospectrometer, with replicates (*n* = 3) stained using Quant-iT™ PicoGreen® dsDNA reagent and measured on the basis of absorbance at 260 nm. The DNA extraction and subsequent quantification method applied here is used comparatively as a general biomass growth indicator, as it does not distinguish between the major groups (Bacteria, Archaea, Eukarya) or between intracellular DNA (from live and dead intact cells) and extracellular DNA (actively released or originating from lysed cells) (Torti et al., 2015).

#### PCR amplification of bacterial 16S rRNA genes

PCR amplifications were carried out using bacterial primers 341GC-F (CGCCCGCCGCGCGCGGCGGGCGGGGCGGGGGCACGGGGGGCCTACGGGAGGCAGCAG) and 534-R (ATTACCGCGGCTGCTGG) (Muyzer et al., 1993) for denaturing gradient gel electrophoresis (DGGE) analysis. All PCR amplifications were performed using a GeneAmp PCR system 9700 thermocycler (Applied Biosystems) as described by Folwell et al. (2016). Thermocycling consisted of 95°C for 5 min followed by 32 cycles of 95°C for 5 s, 55°C for 30 s, 72°C for 30 s, with a final elongation of 72°C for 7 min.

#### DGGE

DGGE analysis of bacterial 16S rRNA gene amplicons was performed using the Bio-Rad D Code system as described by Muyzer et al. (1993), running for 16 h on a gradient of 40–60%, and stained with silver nitrate (Acuña Alvarez et al., 2009).

#### 454 pyrosequencing

The composition of the bacterial communities was assessed from 16S rRNA genes libraries constructed from the DNA extracts of selected samples (+EPS_coll_ treatment replicates at day 0 as representative of the starting community, and replicates of +EPS_coll_, +EPS_HB_, and NoAdd-EPS treatments at day 9; Supplementary Table S1) using ROCHE 454 pyrosequencing technology at the NERC Molecular Genetic Facility at the University of Liverpool as described previously (McKew et al., 2011). The analytical procedure described by Folwell et al. (2016) was used. In brief, any sequences <150 bp in read length, containing errors or with low-quality scores, were removed from analysis. The remaining reads were clustered into operational taxonomic units (OTUs) at 95% similarity level and assigned to a taxonomic group using RDP classifier algorithm (Wang et al., 2007).

### Statistical analysis

All analyses were performed with three replicates for each sampling time. Differences in carbohydrate fractions and inorganic nutrients over time and with respect to treatments were tested by two-way repeated measures analysis of variance (factor time and factor treatment) (ANOVA) follow by Student Newman-Kewls multiple comparison tests when significant differences were found. Differences between degradation rates for DOC, TCHO, and cEPS calculated with both the linear degradation model and the one-G model (with data previously linearized by a *Ln* transform) were tested by comparison of slope by analysis of covariance (ANCOVA). Analyses were performed using the software PAST v 3.10 (Hammer et al., 2001).

Concentration of DNA was *Ln* transformed and fitted to a linear model where slopes represented the net growth. Differences between treatments +EPS_coll_ and +EPS_HB_ were tested by comparison of slopes by ANCOVA.

Changes in the community composition with treatment and over time were analyzed by permutational analysis of variance (PERMANOVA) (Anderson, 2001) on a Bray Curtis similarity resemblance matrix on data normalized by the total number of good reads in each sample. A total of 10,000 unrestricted permutations was set in all tests. When the number of permutations was small (<100), *p*-values were obtained through Monte Carlo random draw from the asymptotic permutation distribution (Anderson and Robinson, 2003). Community data patterns were then represented non-metric Multi-Dimensional Scaling (MDS) using a Bray-Curtis similarity index. Pearson correlation biplots of *log* (*x* + 1) transformed variables were drawn onto the MDS axes to examine their relationship with observed community patterns. All statistical analyses were run using the programs PRIMER 6.0 and PERMANOVA+ (PRIMER-e).

To test significant differences in taxonomic profiles of bacterial community at day 9 from the +EPS_coll_, +EPS_HB_, and NoAdd-EPS treatments, both at Class and at OTU level, we used Whelch's *t*-test implementation of STAMP v2 1.3 package (Parks and Beiko, 2010; Parks et al., 2014) with default parameters except that parameters for filtering out were: *p* > 0.05; difference between proportions < 0.2 or differences between ratios < 1.5.

### Sequence accession

Raw pyrosequences of amplified bacterial 16S rRNA genes from all the samples can be extracted from the European Nucleotide Archive (ENA) under accession number PRJEB15429. Supplementary Table S1 provides the information required to identify the relationship between sample and sequences.

## Results

### Changes in organic carbon and carbohydrate fractions

**Figure 1:**
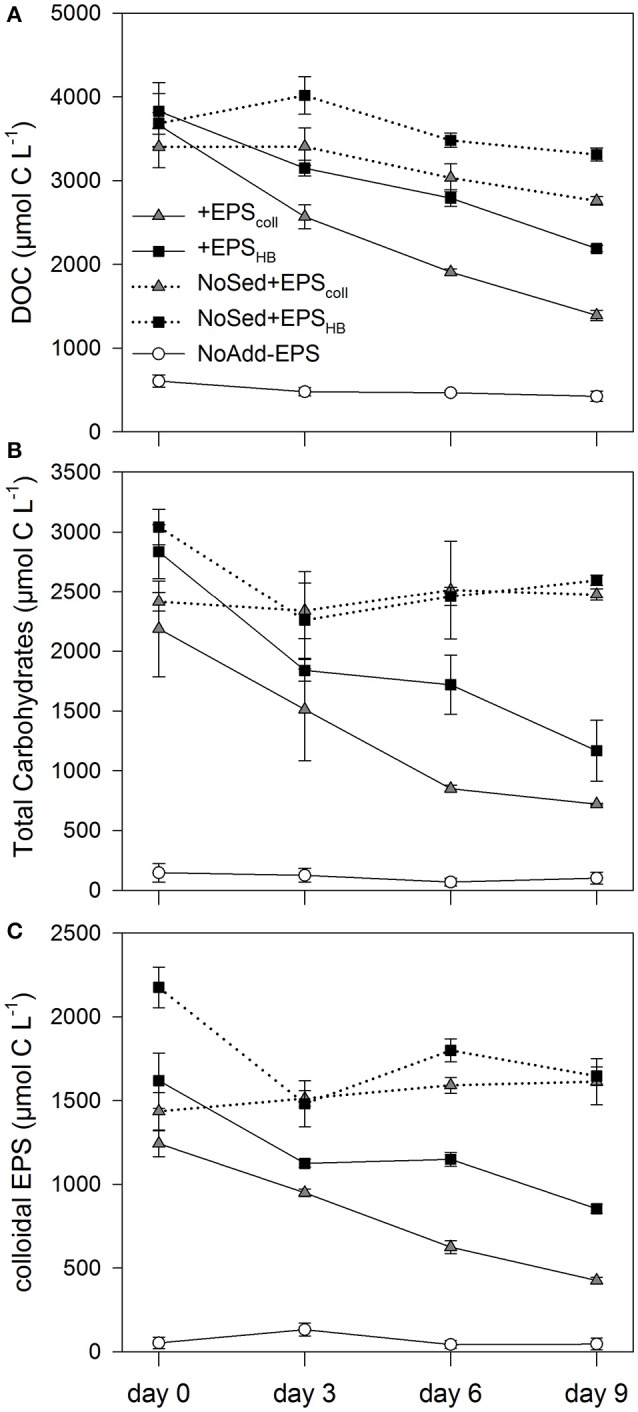
**Concentrations of the three carbohydrate fractions measured: Dissolved Organic Carbon (DOC) (A)**, Total Carbohydrates **(B)** and colloidal EPS **(C)**, in estuarine sediment slurries (200 mg from the top 2 mm, 80 mL artificial sea water (ASW) over 9 days) for the treatments +EPS_coll_, +EPS_HB_, NoAdd-EPS and the controls (no sediment added), NoSed+EPS_coll_ and NoSed+EPS_HB_ represented with dotted lines. Values are means (*n* = 3) ± standard error (SE), expressed as μmol C L^−1^ of slurry.

The addition of EPS resulted in significantly higher DOC concentrations (between 3,600 and 3,800 μmol C L^−1^ on day 0, corresponding to 1.44 and 1.52 mmol C g^−1^ Wet Weight (WW) sediment, respectively) in all four EPS-addition treatments compared with the NoAdd-EPS control (DOC concentration 608 ± 74 μmol C L^−1^ or 0.24 mmol C g^−1^ WW sediment on day 0) (Figure 1A; Student-Newman-Keuls (SNK test), *p* < 0.05). Similarly, the TCHO concentration and cEPS concentration at day 0 (0.8–1.1 mmol C g^−1^ WW sediment and 0.5–0.6 mmol C g^−1^ WW sediment, respectively) were 15–19-fold and 20–30-fold higher, respectively, in EPS-supplemented microcosms compared with the NoAdd-EPS control (Figures 1B,C). The concentrations of DOC, TCHO and cEPS decreased significantly throughout the 9-day experiment in the treatments that had both added EPS and sediment inocula (+EPS_coll_, +EPS_HB_) (Figures 1A–C). However, there were no significant changes in any of the organic carbon fractions in the NoSed+EPS_coll_, NoSed+EPS_HB_, and NoAdd-EPS controls during the experiment (Figures 1A–C).

The value of *k* for the three different carbon fractions (DOC, TCHO, and cEPS) did not differ significantly within treatments, +EPS_coll_ and +EPS_HB_ (Table 1). However, values of *k* for DOC and cEPS fractions in the +EPS_coll_ treatment were significantly higher than those calculated for +EPS_HB_ treatment. Values of *k* for the TCHO fraction between +EPS_coll_ and +EPS_HB_ treatments were not statistically different (Table 1). The linear degradation rates of cEPS in both +EPS_coll_ and +EPS_HB_ treatments were significantly lower than those of DOC and TCHO (*p* < 0.05, ANCOVA).

**Table 1:** **Comparison of degradation constant (***k***) calculated using the one-G model for degradation of organic matter (Berner, **1964**) or the lineal degradation constant (***b***) for the three carbon fractions measured [Dissolved Organic Carbon (DOC), Total Carbohydrates (TCHO) and colloidal EPS (cEPS)] in the +EPS_**coll**_ and +EPS_**HB**_ treatments during the 9-day period (± standard error of ***k*** and ***b***)**.

**Fraction**	+**EPS_coll_**		+**EPS_HB_**	
	***k* (d^−1^)**	***b* (mg C_org_ g^−1^ sed. WW d^−1^)**	***k* (d^−1^)**	***b* (mg C_org_ g^−1^ sed. WW d^−1^)**
Dissolved Organic Carbon (DOC)	0.105^a^ ± 0.01	1.19 ± 0.18	0.060^b^ ± 0.005	0.84^a^ ± 0.08
Total Carbohydrates (TCHO)	0.123^a^ ± 0.02	0.81^a, b^ ± 0.19	0.096^a, b^ ± 0.02	0.68^a^ ± 0.16
Colloidal EPS (cEPS)	0.121^a^ ± 0.007	0.44^b^ ± 0.03	0.062^b^ ± 0.01	0.36^b^ ± 0.07

*The determination coefficients were significant for both the exponential and the lineal fitting (p < 0.05, n = 12). Differences in k or b were compared by ANCOVA between organic fractions (DOC, TCHO and cEPS) within the same treatment (+EPS_coll_ or +EPS_HB_), and between treatments for the same organic fraction. The same superscript letter (a,b) means absence of statistically significant differences among the corresponding values of k or b (p < 0.05)*.

The lower value of *k* for the three carbon fractions in the +EPS_HB_ treatment indicated a lower degradability that must be based in chemical differences between these fractions derived from the EPS_coll_ or the EPS_HB_ enrichments. To address possible changes in the overall chemical composition in the added organic fractions, EPS_coll_ or the EPS_HB_, we calculated the TCHO:DOC, cEPS:DOC, and cEPS:TCHO mass ratios in both enrichments (Supplementary Figure S1). These ratios were similar at day 0 for both enrichments, with observed differences not statistically significant. In general, both TCHO:DOC and cEPS:DOC remained constant or gradually decreased during the experiment in both enrichments. On the contrary cEPS:TCHO ratio in the EPS_HB_ enrichment increased significantly (linear correlation; *r*^2^ = 0.9664; *p* < 0.02) with time, suggesting a lower relative degradability of cEPS material within the +EPS_HB_ enrichment.

### Changes of dissolved inorganic nutrients

**Figure 2:**
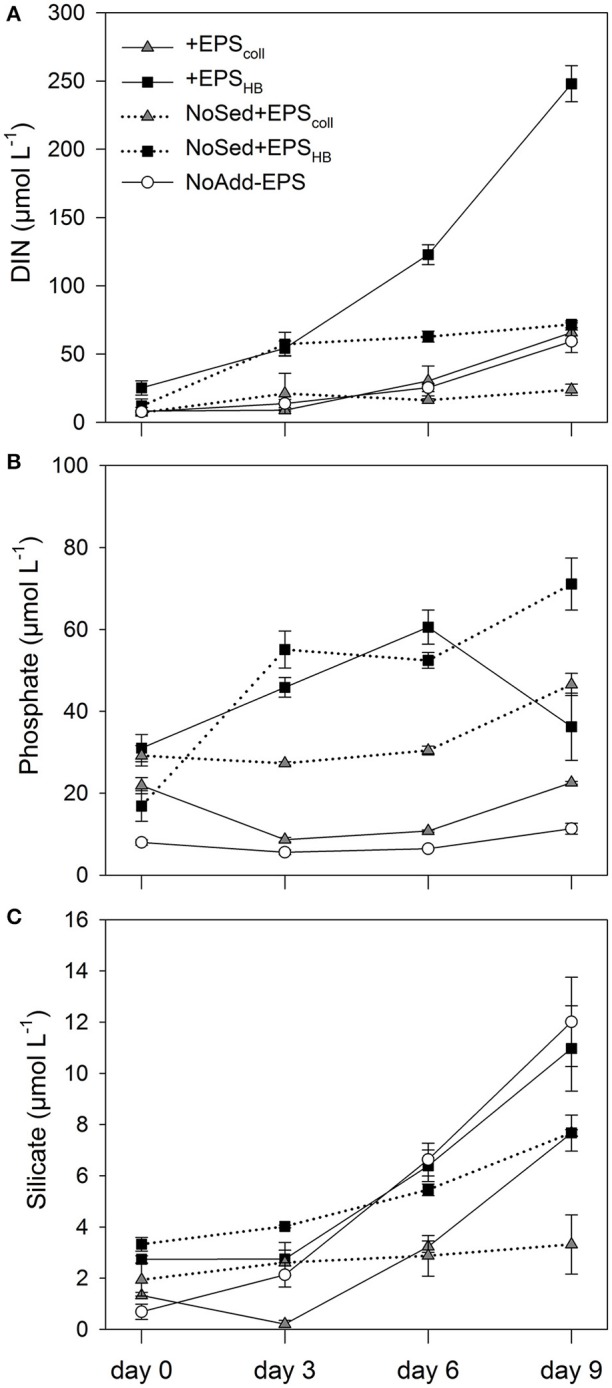
**Concentrations of different inorganic nutrients measured: Nitrate, Nitrite and Ammonium expressed as Dissolved Inorganic Nitrogen (DIN) (A)**, Phosphate **(B)**, and Silicate **(C)** in estuarine sediment slurries (200 mg from the top 2 mm, 80 mL artificial sea water (ASW) over 9 days) for the treatments +EPS_coll_, +EPS_HB_, NoAdd-EPS and the controls (no sediment added), NoSed+EPS_coll_ and NoSed+EPS_HB_ represented with dotted lines. Values are means (*n* = 3) ± standard error (SE), expressed as μmol L^−1^ of slurry.

The EPS_coll_ and EPS_HB_ fractions added to the enrichments and the sediment inoculum contributed to the initial concentration of dissolved inorganic nutrients measured in the different treatments. These concentrations of nutrients, directly added with the EPS and sediment inoculum, were relatively low for all investigated nutrients at day 0 (${\text{NO}}_{3}^{-}$ + ${\text{NO}}_{2}^{-}$ < 6.5 μmol L^−1^, ${\text{NH}}_{4}^{+}$ < 18.8 μmol L^−1^, ${\text{PO}}_{4}^{3-}$ < 31.0 μmol L^−1^, and ${\text{SiO}}_{4}^{4-}$ < 2.3 μmol L^−1^). The general trend was an increase of all nutrients in all treatments with time (Figure 2). This increase over time is necessarily the result of the net regeneration of inorganic nutrients from the mineralization of the EPS fractions and/or the organic matter introduced with the sediment inoculum.

Concentrations of Dissolved Inorganic Nitrogen (DIN, the sum of nitrate, nitrite and ammonium concentrations) increased steeply with time in +EPS_HB_ treatment(24.6 μmol L^−1^ day^−1^), while the regeneration rates were significantly lower (SNK test, *p* < 0.05) in the rest of the treatments (1.5–6.5 μmol L^−1^ day^−1^). There were no significant differences in DIN regeneration rates between +EPS_coll_ and NoAdd-EPS treatments that were 6.5 and 5.5 μmol L^−1^ day^−1^, respectively (Figure 2A). The mineralization of the EPS_HB_ fraction was a major source of DIN regeneration, initially in the form of ${\text{NH}}_{4}^{+}$ and later in the form of ${\text{NO}}_{3}^{-}$ + ${\text{NO}}_{2}^{-}$ (${\text{NO}}_{\text{x}}^{-}$) (Supplementary Figure S2). The importance of the net regeneration of ammonium and ${\text{NO}}_{\text{x}}^{-}$ shifted during the experiment as shown by changes in ${\text{NO}}_{\text{x}}^{-}$: ${\text{NH}}_{4}^{+}$ ratio, which increased from about 0.15 at day 3 up to 3.3 at day 9. The regeneration rate of ${\text{NH}}_{4}^{+}$ at the beginning of the experiment was similar in both +EPS_HB_ treatment and the +EPS_HB_ control without sediment (8.6–9.6 μmol L^−1^ day^−1^) and considerably higher than in the other treatments (1.8–3.3 μmol L^−1^ day^−1^). This emphasizes the strong difference between EPS_HB_ and EPS_coll_ regarding their potential as sources of regenerated inorganic nitrogen.

The EPS_HB_ fraction was a major source of regenerated dissolved phosphate, since its concentration increased in both treatments with added EPS_HB_ (+EPS_HB_ and NoSed+EPS_HB_) at a similar rate (4.9–5.9 μmol L^−1^ day^−1^) up to the day 6 (Figure 2B). Dissolved phosphate decreased significantly between days 6 and 9 in the +EPS_HB_ treatment (SNK, *p* < 0.05) but not in the NoSed+EPS_HB_ control. On the contrary, phosphate changed little or even decreased during the experiment in treatments with EPS_*coll*_ or sediment only.

Silicate concentrations increased with time, particularly from day 3 onwards in all of the treatments with added sediment, +EPS_coll_, +EPS_HB_, and NoAdd-EPS treatments (Figure 2C). The regeneration of silicate was positively affected by the sediment inoculum, since it was higher in all the treatments with sediment (0.7–1.28 μmol L^−1^ day^−1^) compared with NoSed+EPS_coll_ and NoSed+EPS_HB_ controls lacking sediment inoculum, 0.16 and 0.41 μmol L^−1^ day^−1^, respectively. This likely indicates that the sediment inoculum was the source of regenerated silicate during the experiment and not the added EPS.

### Changes in DNA concentration of sediment slurries

**Figure 3:**
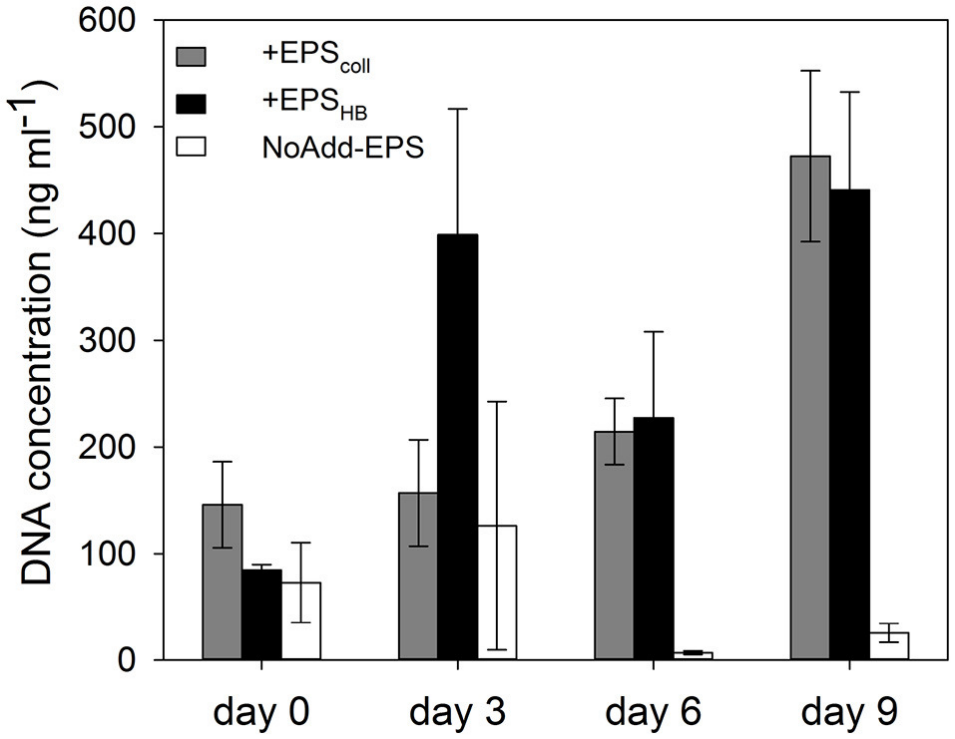
**DNA concentration (as a proxy for biomass) in estuarine sediment slurries for the treatments +EPS_**coll**_, +EPS_**HB**_, and NoAdd-EPS, expressed as ng mL^**−1**^ of slurry**.

Extracted DNA concentration was used as a proxy for microbial biomass (Figure 3). At the beginning of the experiment, there were no significant differences between DNA concentrations in the +EPS_coll_, +EPS_HB_, and NoAdd-EPS treatments (Figure 3). DNA concentration increased significantly (by around 400%) in the +EPS_coll_, and +EPS_HB_ treatments by the end of the experiment (SNK, *p* < 0.05). In the NoAdd-EPS sediment-only control DNA concentration decreased by day 9 compared to day 0, with concentrations significantly lower than in the organic-carbon-amended treatments (+EPS_coll_ and +EPS_HB_) (SNK, *p* < 0.001). Microbial biomass exhibited exponential-like growth in the treatments +EPS_coll_ and +EPS_HB_ (*r*^2^ = 0.84 and *r*^2^ = 0.56, respectively), and no significant differences were observed between these two treatments.

### Changes in the bacterial community composition

Denaturing gradient gel electrophoresis (DGGE) of partial bacterial 16S rRNA genes (Supplementary Figure S3) showed consistency in the banding patterns between replicate treatments at day 9, and showed that the day-0 communities were identical regardless of treatment, justifying the use of the day-0 +EPS_coll_ treatment only in subsequent pyrosequence analysis. By day-9 the bacterial community composition had changed from day-0 even in the NoAdd-EPS control, and at day-9 there were distinct differences in bacterial community composition between the treatments +EPS_coll_, +EPS_HB_, and NoAdd-EPS.

**Figure 4:**
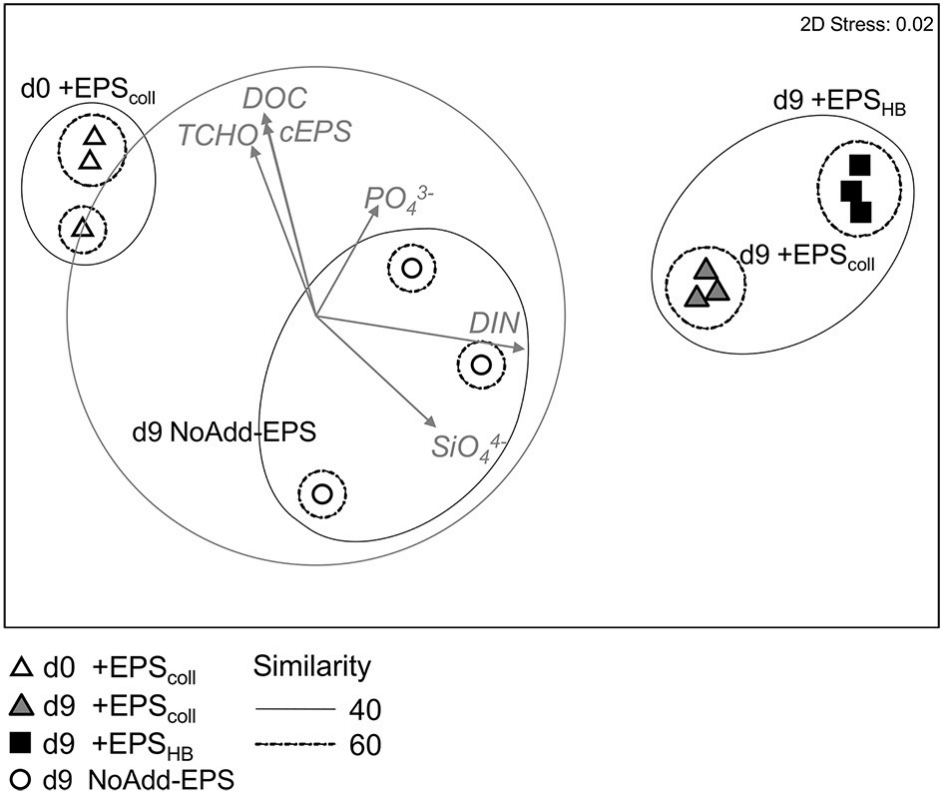
**Non-metric Multi-Dimensional Scaling ordination plot based on Bray Curtis similarity of relative abundance data of the bacterial community from the different treatments +EPS_**coll**_, +EPS_**HB**_, and NoAdd-EPS at day 9**. Samples of +EPS_coll_ treatment at day 0 are used as initial samples for the rest of treatments (stress: 0.02). Points represent centroids of replicate samples. Groups at 40 and 60% similarity are shown after applying a group average clustering. The vector overlay shows the environmental variables with correlation >0.5. Arrows indicate direction and relative magnitude of influence.

In order to quantify the changes in bacterial community composition and identify key taxa putatively involved in EPS degradation, pyrosequencing of 16S rRNA genes was performed on +EPS_coll_, +EPS_HB_ and NoAdd-EPS treatments after 9 days, and from a representative day-0 sample, +EPS_coll_. Multidimensional scaling analysis revealed statistically significant temporal- and treatment-related changes (Figure 4). The bacterial communities from day-9 +EPS_coll_ and +EPS_HB_ treatments were different from both the NoAdd-EPS treatment at day 9 and the starting community (*p* < 0.05) (Figure 4). After 9 days, the NoAdd-EPS community was more similar to the starting community than those with added EPS. Also, there was a significant difference (*p* < 0.05) in the bacterial community composition between the +EPS_coll_ and +EPS_HB_ treatments at day 9.

**Figure 5:**
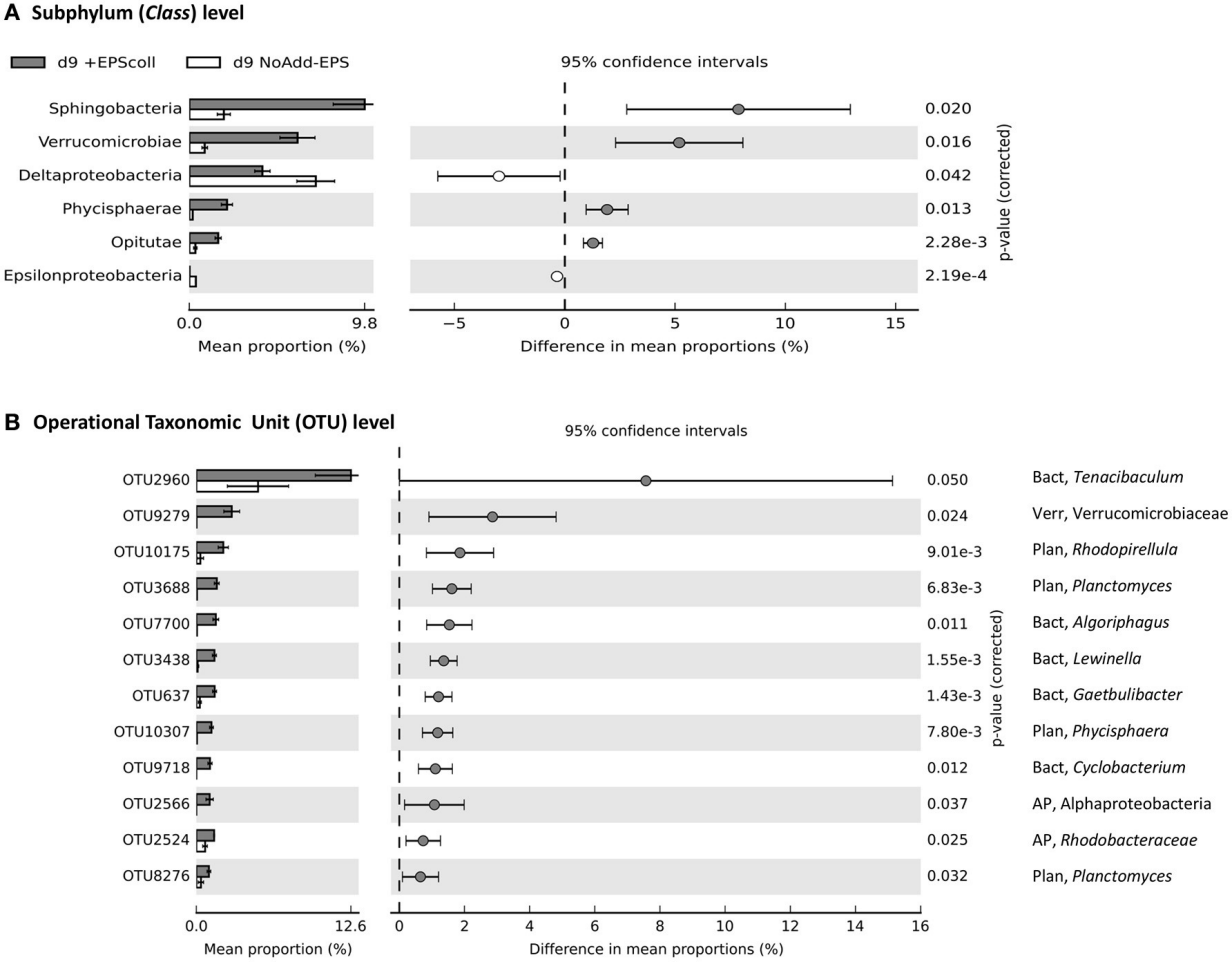
**Comparison of bacterial community profiles in +EPS_**coll**_ vs. NoAdd-EPS by day 9 both at the levels of subphylum (class) (A)** and Operational Taxonomic Unit (OTU) defined at >95% similarity **(B)**. Analysis was performed using STAMP (Parks and Beiko, 2010; Parks et al., 2014) with default parameters except that parameters for filtering out were: *p* > 0.05; difference between proportions <0.2 or difference between ratios <1.5. Data were sorted according to effect size. Note the differences in the scale of the x axes. The only phyla that were significantly enriched at day-9 in EPS_*coll*_ treatment compared with the NoAdd-EPS control were the Bacteroidetes (~2-fold more abundant) and Verrucomicrobia (~5-fold more abundant; data not shown). The information to the right of the *p*-values is the identity of the OTU, whereby the phylum is indicated to the left of the comma (AP, Alphaprotebacteria; Bact, Bacteroidetes; Plan, Planctomycetes; Verr, Verrucomicrobia), and the lowest taxonomic level to which the OTU can be confidently assigned is indicated to the right of the comma. A total of 23 bacterial taxa was significantly enriched in +EPS_*coll*_ treatment compared with NoAdd-EPS treatment but only the top 12 is shown.

The only phyla that were significantly enriched at day-9 in the +EPS_coll_ treatment compared with the NoAdd-EPS control were the Bacteroidetes (~2-fold more abundant) and Verrucomicrobia (~5-fold more abundant; data not shown). These enrichments were specifically within the Sphingobacteria (Bacteroidetes), two classes from Verrucomicrobia (Verrucomicrobiae and Opitutae) and one class of Planctomycetes (Phycisphaerae), which were all significantly more abundant in the +EPS_coll_ treatment (Figure 5A; see figure for statistical criteria). At the level of operational taxonomic units clustering at 95% similarity (OTU_95_), OTU-2960, from the genus *Tenacibaculum* in the phylum Bacteroidetes, had the biggest additive increase in the treatment +EPS_coll_ (12% relative abundance) compared with NoAdd-EPS (5%; Figure 5B). The Verrucomicrobia OTU-9279 increased most in relative abundance, constituting 3% of the community in treatment +EPS_coll_ while in the NoAdd-EPS control it was absent (Figure 5B).

**Figure 6:**
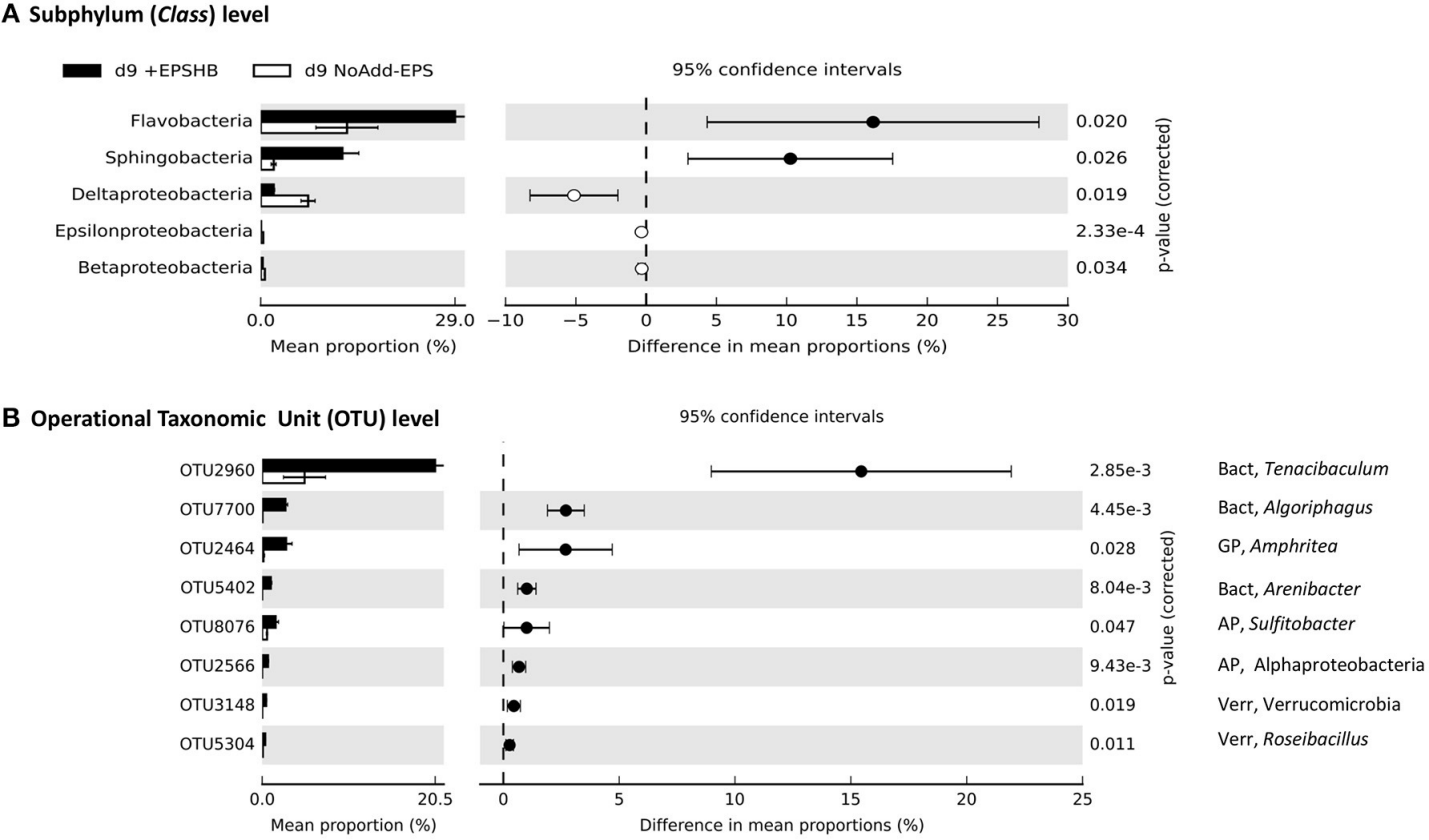
**Comparison of bacterial community profiles in +EPS_**HB**_ vs. NoAdd-EPS by day 9 both at the levels of subphylum (class) (A)** and Operational Taxonomic Unit (OTU) defined at >95% similarity **(B)**. Analysis was performed using STAMP (Parks and Beiko, 2010; Parks et al., 2014) with default parameters except that parameters for filtering out were: *p* > 0.05; difference between proportions <0.2 or difference between ratios <1.5. Data were sorted according to effect size. Note the differences in the scale of the x axes. The only phyla that were significantly enriched at day-9 in EPS_HB_ treatment compared with the NoAdd-EPS control were the Bacteroidetes (~2.5-fold more abundant) and Verrucomicrobia (~6-fold more abundant; data not shown). The information to the right of the *p*-values is the identity of the OTU, whereby the phylum is indicated to the left of the comma (AP, Alphaprotebacteria; Bact, Bacteroidetes; GP, Gammaproteobacteria; Verr, Verrucomicrobia), and the lowest taxonomic level to which the OTU can be confidently assigned is indicated to the right of the comma. A total of eight bacterial taxa were significantly enriched in +EPS_HB_ treatment compared with NoAdd-EPS treatment.

Bacteroidetes and Verrucomicrobia were also significantly enriched at day-9 in the +EPS_HB_ treatment compared with the NoAdd-EPS control (~2.5-fold and ~6-fold more abundant respectively; data not shown). At the subphylum level, only Flavobacteria and Sphingobacteria from the Bacteroidetes were enriched significantly (Figure 6A). As with the +EPS_coll_ treatment, there was a big increase in OTU-2960 from the genus *Tenacibaculum* (phylum Bacteroidetes) comprising 20% of the relative abundance (Figure 6B). OTU-7700, with 100% identity to *Algoriphagus yeomjeoni* from the phylum Bacteroidetes and OTU-2464 from the gammaproteobacterial genus *Amphritea*, both had the biggest relative increase after EPS_HB_ addition, and comprised ~3% of the day-9 +EPS_HB_ community (Figure 6B).

Bacterial communities from +EPS_coll_ and +EPS_HB_ treatments were 52% similar but significantly different (Figure 4, *p* < 0.05). There were no phylum-level significant differences in abundance between these two treatments. However, the addition of EPS_coll_ stimulated significant increases in Opitutae from the phylum Verrucomicrobia and in Deltaproteobacteria as the only identified classes compared to EPS_HB_-amended treatment (+EPS_HB_ treatment) (Supplementary Figure S4A). Three aforementioned OTUs belonging to the genera *Tenacibaculum, Amphritea*, and *Algoriphagus* were significantly relatively more abundant in +EPS_HB_, whereas a range of OTUs from diverse phyla was relatively more abundant in +EPS_coll_ (Supplementary Figure S4B).

### Relationship between the bacterial community composition and environmental variables

Pearson correlation biplots drawn on the MDS showed that DIN correlated strongly with the horizontal axis, separating the day 9 bacterial communities of +EPS_coll_ and +EPS_HB_ treatments from the initial samples. cEPS and the rest of the organic carbon source related variables (TCHO; DOC) had a high correlation with the second axis, which separates both +EPS_coll_ and +EPS_HB_ treatments from NoAdd-EPS treatment.

## Discussion

### Degradation of colloidal EPS by heterotrophic bacteria

The degradation of organic matter is a chemically and microbiologically complex process because organic matter is typically a mixture of organic compounds with different relative degradability (Hedges and Oades, 1997; Burdige, 2007; Arndt et al., 2013). Microbial degradation involves the extracellular breakdown of HMW polymers to LMW oligosaccharides and monomers (Goto et al., 2001; Hofmann et al., 2009), which can be readily incorporated by bacterial cells, with the initial polymer hydrolysis generally being the rate-limiting step (Meyer-Reil, 1990; Kristensen and Holmer, 2001). In our experiment, addition of EPS stimulated the degradation of the organic fractions (DOC, TCHO, and cEPS) in both +EPS_coll_ and +EPS_HB_ treatments (Figure 1). Ratios of cEPS:DOC and TCHO:DOC decreased consistently in both treatments, which might indicate a preferential degradation of cEPS and TCHO compared to DOC. On the other hand, the difference in the time evolution of cEPS:TCHO ratios between treatments suggests a lower degradability of cEPS from the hot-bicarbonate fraction. Typically, most slurry studies have found that the hot-bicarbonate carbohydrate fraction is more refractory that the cEPS fraction in oxic conditions (Table 2).

**Table 2:** **Comparison of degradation rates for different carbon fractions from previous published studies**.

**Publication**	**Added DOM/EPS**	**Fraction measured**	**Starting Conc**.	**Rate of degradation (units: ^§^ = μg gluc. g^−1^ DW d^−1^; φ = μg g^−1^ DW biofilm h^−1^; ¤ = μg gluc. g^−1^ WW h^−1^; † = μg C g^−1^ DW d^−1^)**	
Goto et al., 2001				^14^C-labeled MPB community	
	^14^C-EDOC	^14^C-EDOC	NP^*^	*k* (d^−1^): 0.616	
	^14^C-EDTA-OC	^14^C-EDTA-OC	NP^*^	*k* (d^−1^): 0.916	
	^14^C-EPS_coll_	^14^C-EPS_coll_	NP^*^	*k* (d^−1^): 0.616	
Haynes et al., 2007^b^	CHO_coll_	EPS_coll_ (μg gluc. g^−1^ DW)	233.8	0–2 d: −81 ^§^	4–10 d: +50 ^§^
		CHO_HB_ (μg gluc. g^−1^ DW)	336.5	NP	
	EPS_coll_	EPS_coll_ (μg gluc. g^−1^ DW)	383.2	0–2 d: −140 ^§^	4–10 d: +224.8 ^§^
		CHO_HB_ (μg gluc. g^−1^ DW)	482.7	NP	
Hofmann et al., 2009^c^	CHO_coll_	EPS_coll_ (μg gluc. mL^−1^ slurry)	10.2	0–4 h: +15 ¤	4–24 h: −3.5 ¤
	EPS_coll_	EPS_coll_ (μg gluc. mL^−1^ slurry)	27.7	0–4 h: −81 ¤	4–24 h: +0.85 ¤
Bellinger et al., 2009^d^	None^**^	Control treat			
		CHO_HB_ (μg g^−1^ DW biofilm)	1,625	4–12 h: +16.37 φ	
		13C-enriched treat			
		CHO_HB_ (μg g^−1^ DW biofilm)	1,300	4–12 h: +3.8 φ	12–48 h: −3.9 φ
Oakes et al., 2010^a^	None^**^			First order decay rate (2-G model)	
		Mannose (mmol C m^−2^)	24.8	*k*_1_ (d^−1^): 4.38 ± 1.02	*k*_2_ (d^−1^): 0.01 ± 0.01
		Fucose (mmol C m^−2^)	49.2	*k*_1_ (d^−1^): 1.5 ± 0.29	*k*_2_ (d^−1^): 0.05 ± 0.01
		Rhamnose (mmol C m^−2^)	80	*k*_1_ (d^−1^): 0.81 ± 0.18	*k*_2_ (d^−1^): 0.03 ± 0.01
		Galactose (mmol C m^−2^)	105	*k*_1_ (d^−1^): 1.27 ± 0.25	*k*_2_ (d^−1^): 0.03 ± 0.01
		Glucose (mmol C m^−2^)	113	*k*_1_ (d^−1^): 1.38 ± 0.25	*k*_2_ (d^−1^): 0.04 ± 0.01
		Xylose (mmol C m^−2^)	34.2	*k*_1_ (d^−1^): 1.43 ± 0.34	*k*_2_ (d^−1^): 0.02 ± 0.02
McKew et al., 2013^e^	None	DOC (μg C g^−1^ DW)	565	Aer. 0–10 d: −44 †	Aer. 10–25 d: +6 †
				Anaer. 0–10 d: −36 †	Anaer. 10–25 d: +7 †
		CHO_HB_ (μg C g^−1^ DW)	488	Aer. 0–10 d: +12 †	Aer. 10–25 d: −8.8 †
				Anaer. 0–10 d: −6 †	Anaer. 10–25 d: +1 †
		TCHO (μg C g^−1^ DW)	2,894	Aer. 0–10 d: −99 †	Aer. 10–25 d: +9.8 †
				Anaer. 0–10 d: −100 †	Anaer. 0–10 d: +3.6 †
Taylor et al., 2013^f^	^13^C-labeled EPS	CHO_coll_ (ug gluc. g^−1^ WW)	89.85	0–30 h: +0.07¤	30–72 h: −1.06 ¤
		EPS_coll_ (ug gluc. g^−1^ WW)	47.75	0–30 h: −0.7 ¤	30–72 h: −0.2 ¤
		DOC in CHO_coll_ (ug gluc. g^−1^ WW)	402.23	0–72 h: −3.4 ¤	
		DOC in EPS_coll_ (ug gluc. g^−1^ WW)	49.98	0–72 h: −0.5 ¤	
	^12^C-unlabeled EPS	CHO_coll_ (ug gluc. g^−1^ WW)	92.75	0–30 h: +0.39 ¤	30–72 h: −1.2 ¤
		EPS_coll_ (ug gluc. g^−1^ WW)	48.27	0–30 h: −1.03 ¤	
		DOC in CHO_coll_ (ug gluc. g^−1^ WW)	394.2	0–72 h: −2.9¤	
		DOC in EPS_coll_ (ug gluc. g^−1^ WW)	41.22	0–72 h: −0.24 ¤	
Miyatake et al., 2014^g^	None^**^	TCHO (μmol C g^−1^ DW)	27.1	NSC (27.04 μmol C g^−1^ DW; max. of 29.5 at 72 h)	
		CHO_coll_ (μmol C g^−1^ DW)	3.6	0–72 h: −0.04 (μmol C g^−1^ DW h^−1^; incr. of 1.9 at 120 h)	
		EDTA-CHO (μmol C g^−1^ DW)	0.85	NSC (0.99 μmol C g^−1^ DW; min. of 0.3 at 72 h)	

*Rates were calculated using the data presented in the paper in the form of tables or graphs, when not provided by the authors. In the final column, showing the rates of degradation, sub-columns are used to indicate the rates with different treatments as indicated. In order to avoid confusion, rates are presented in the units given in the original publication. Symbols + and − preceding the degradation rates indicate accumulation or loss, respectively. Abbreviations and details of the microbial community involved in the degradation of the added carbon source are provided in the footnote*.

* Although starting concentrations were not provided, calculations for degradation rates were estimated using the percentage of added substrates mineralized.

** *In these experiments no additional organic carbon source was added; sediment DOC/EPS was labeled using NaH^13^CO_3_*.

Details about the microbial community composition and EPS-induced changes are as follows:

a *Microbial community was not studied*.

b *Increases in Bacteroidetes and Alphaproteobacteria, especially Acinetobacter (and in particular in the cEPS-enrichment)*.

c *Isolation of Variovorax sp. as the main EPS-degrader (Betaproteobacteria)*.

d *Gram-negative bacteria incorporated diatom-derived carbon faster than Gram-positive bacteria*.

e *Aerobic slurries had a high relative abundance of Gammaproteobacteria and a big increase in Verrucomicrobia, while in the anaerobic slurries Deltaproteobacteria were dominant (especially Desulfobacteraceae and Desulfobulbaceae)*.

f *Alphaproteobacteria and Gammaproteobacteria increased in the slurry with added ^13^C-diatom derived EPS*.

g *The bacterial community was dominated by Gammaproteobacteria (21%), and to a lesser extent by Bacteroidetes (8%) and Deltaproteobacteria (7%)*.

*Abbreviations of DOM/EPS sources are: DOM, Dissolved Organic Matter; EPS, Extracellular Polymeric Substances; ^14^C-EDOC, ^14^C-labeled Extracellular Dissolved Organic Carbon; ^14^C-EDTA-OC, ^14^C-labeled EDTA-extracted Organic Carbon; ^14^C-EPS_coll_, ^14^C-labeled colloidal EPS; CHO_coll_, colloidal Carbohydrates; CHO_HB_, Hot-Bicarbonate-extracted Carbohydrates; DOC, Dissolved Organic Carbon; TCHO, Total Carbohydrates; EDTA-CHO, EDTA-extracted Carbohydrates. Other abbreviations are: NP, Non-provided; NSC, No significant changes*.

Organic matter degradation in marine sediments usually follows an exponential decay with time described by the G-models family (Berner, 1964; Arndt et al., 2013). Only a few studies have applied this model in EPS-related studies. Oakes et al. (2010) studying the degradation rates of several monosaccharide pools applied a 2-G model which assumes that the pool of organic matter consists of two fractions that degrade exponentially at different rates and included a non-reactive fraction as well. Three fractions in each monosaccharide pool were detected: (1) a highly labile fraction accounting for the largest part (65–87%) of each monosaccharide pool, with high exponential decay rates (*k*) (0.81–4.38 d^−1^); (2) a more refractory fraction (7–18% of each monosaccharide pool), whose *k* was one or two order of magnitude lower (0.01–0.07 d^−1^); and (3) a non-reactive fraction (6–23% of each monosaccharide pool) (Table 2). The presence of a second most refractory component was evident in the degradation kinetics of various sediment-extracted carbohydrate fractions both in oxic and anoxic conditions in a 25-day slurry experiment (McKew et al., 2013). Although the loss of TCHO and cEPS over time in our experiment could suggest the existence of a second more refractory component (especially in the +EPS_HB_ treatment), testing for the inclusion of such a component in the model did not provide a significantly better fit (results not shown). It is probable that the time scale of our experiment was too short to detect the existence of more than one pool in the degradation kinetics of every fraction.

The exponential decay rates in our experiment (Table 1) fall between those of the highly reactive fraction and the less reactive fraction of Oakes et al. (2010) for specific monosaccharides. However, the exponential decay rates from the current study for specific carbohydrates and more complex or less unambiguously defined carbohydrate-related fractions, like TCHO and EPS, extracted from microbenthic algae (this study, Goto et al., 2001; Oakes et al., 2010), span two orders of magnitude (Table 2). Interestingly, these rates are higher and less variable than the wider range of reported *k*-values (10^−11^–10^−2^ d^−1^) for the degradation of bulk organic matter in different marine sediments (Arndt et al., 2013). This indicates the general lability of diatom-biofilm EPS in comparison to detritus derived from other sources, and highlights its importance in structuring heterotrophic communities (Hofmann et al., 2009; Taylor et al., 2013). However, comparison of degradation rate constants between different experiments or different environments must be done with caution since the degradability of organic matter depends on the interaction of its chemical composition and the particular environmental conditions where degradation takes place (Mayer, 1995; McKew et al., 2013).

### EPS degradation and inorganic nutrient regeneration

Nutrient concentrations detected on day 0 were higher in all treatments with added EPS compared to the control. Although the sediment inoculum added may have represented a small source of inorganic nutrients to the slurries, it seems that both EPS-extraction methods recover some dissolved inorganic nutrients from the sediment plus microphytobenthic biofilm samples, a source of nutrients not previously accounted for (Figure 2). The extraction protocol of both EPS fractions involves freezing the sediment sample, which is known to break algal and bacterial cells releasing relatively large amounts of intracellular dissolved inorganic nutrients (García-Robledo et al., 2010, 2016; Stief et al., 2013; Yamaguchi et al., 2015). Although, there were no significant differences on day 0 between nutrients in the +EPS_coll_ and +EPS_HB_ treatments, the hot-bicarbonate method extracted an organic matter pool that was particularly rich in organic N and P (Figure 2, Supplementary Figure S2). Increases in dissolved inorganic nutrients during the experiment were more pronounced and rapid with added EPS_HB_ than for +EPS_coll_, and represent the mineralization of nutrients associated to organic compounds included in the EPS_HB_ fraction. EPS_HB_ is one of the more abundant and heterogeneous fractions (Chiovitti et al., 2003) the extraction procedure of which not only recovers the extracellular carbohydrates but also uronic acids, proteins, glycoproteins, and phospholipids tightly bound to the mucilage of the diatom frustules (Underwood et al., 1995; Wustman et al., 1997).

The regeneration rate of ${\text{NH}}_{4}^{+}$ was similar in both treatments with EPS_HB_, with and without sediment, suggesting a wider distribution of this trait such that the presence of the sediment microbial community plays a minor role in this process. On the contrary, further transformation of ${\text{NH}}_{4}^{+}$ to ${\text{NO}}_{2}^{-}$ and ${\text{NO}}_{3}^{-}$, detected in the +EPS_HB_ treatment, particularly toward the end the experiment (Figure 2A, Supplementary Figure S2B), can be only explained by an increase of nitrification rates due to the growth of a community of nitrifiers introduced with the sediment inoculum, that was absent in the control without sediment. Nitrifying bacteria were indeed present in our samples (e.g., *Nitrospira*, which oxidizes nitrite to nitrate, and the ammonia-oxidizing genus *Nitrosospira*), albeit in low relative abundance (~0.01%) in the +EPS_HB_ treatment.

EPS_HB_ was also an important source of regenerated ${\text{PO}}_{4}^{3-}$. The initial stoichiometry between ${\text{NH}}_{4}^{+}$ and ${\text{PO}}_{4}^{3-}$ regeneration rates was about 1.7 in both EPS_HB_ treatments, considerably richer in P than typical microphytobenthic biomass (Hillebrand and Sommer, 1999). MPB biofilm EPS_HB_ extractions do frequently include some ribose, indicating some DNA/RNA contamination (Bellinger et al., 2005, 2009), which may be the source of the phosphate. The decrease of ${\text{NH}}_{4}^{+}$ and ${\text{PO}}_{4}^{3-}$ from day 6 to 9 in the +EPS_HB_ treatment might be explained by higher microbial consumption rate at the end of the experiment. Nonetheless, growth of the microbial community, as estimated from the increase in DNA over 9 days, was similar in both +EPS_HB_ and +EPS_coll_ treatments. Therefore, even the lower amount of regenerated nutrient released from the EPS_coll_ fraction was enough to support the microbial demand for N and P.

The regeneration rate of silicate was 3–4 times higher in the treatments with sediment-inoculum added compared to the controls without sediment inoculum (Figure 2C). Most likely the increase in silicate is mainly due to its regeneration from a particulate pool bound to sediment particles. Even in the absence of any added EPS, in the NoAdd-EPS treatment (with sediment), the regeneration of silicate was similar to that of the +EPS_HB_ treatment with sediment. Therefore, EPS seems to play a minor role in the recycling of silicate in marine sediments in contrast to what we have shown for N and P.

### EPS-induced changes in the bacterial community composition

Incubation of sediment slurries with both types of EPS resulted in a significant increase in microbial biomass by the end of the experiment using total DNA concentration as a proxy (Figure 3; Haynes et al., 2007). It also led to a significant shift in community composition, both temporally and as direct result of EPS addition (Figure 4). Given that EPS was the differentially added component, constituting a high proportion of the available DOC, and that its degradation was ongoing at day 9, the selectively enriched and actively growing microbial community at this time would contain a high proportion of active EPS-degraders. However, the dominance of such bacterial taxa is also the net result of the growth at the expense of the added EPS sources and any losses by grazing and viral lysis (Våge et al., 2013; Thingstad et al., 2014), which might have increased due to the increased inorganic nutrient content in the slurries (Miki and Jacquet, 2010). The relative similarity of many of the taxa comprising the bacterial communities in the +EPS_coll_ and +EPS_HB_ treatments indicate that most EPS-degrading bacteria readily consume a wide range of diatom-derived EPS (Taylor et al., 2013). Nevertheless, the significant difference overall between communities fed with EPS_coll_ and EPS_HB_ indicates that there are a number of specialist bacteria that preferentially use a particular fraction.

Several bacterial phyla, namely Bacteroidetes, Verrucomicrobia and Planctomycetes, increased their presence in the +EPS_coll_ compared with the NoAdd-EPS treatment, and were thus probably involved in EPS_coll_ biodegradation (Figure 5A). Verrucomicrobia is a phylum that is widely distributed, but rarely dominant (Freitas et al., 2012; Yilmaz et al., 2016). As there are few cultivated representatives of this group, we have a poor understanding of its ecophysiology, but evidence is emerging that many species of Verrucomicrobia consume algal EPS and other biopolymers (Martinez-Garcia et al., 2012; Cardman et al., 2014; Landa et al., 2014; Orsi et al., 2016). McKew et al. (2013) showed that, in a mudflat enrichment, Verrucomicrobia had the biggest proportional increase (~6-fold) when incubated aerobically with cEPS, but were ~40% less abundant when grown anaerobically. In addition, PiCrust analysis suggests that Verrucomicrobia have a similar range and number of extracellular enzymes for breaking down complex polymers as do the Bacteroidetes, a recognized major biopolymer-degrading group (Yilmaz et al., 2016). Specifically, a metagenome from the marine *Candidatus* Spartobacteria baltica was rich in glycoside hydrolases (Herlemann et al., 2013). Thus, a picture is emerging that aerobic biopolymer degradation is a key trait of many Verrucomicrobia. Verrucomicrobia were also significantly enriched in the presence of EPS_HB_, suggesting that they are able to degrade complex polymers. Two OTUs had a marginal increase in abundance in the presence of EPS_HB_, one of which was related to *Roseibacillus*, species of which have been isolated from brown algae and also from marine water and sediments (Yoon et al., 2008), which may further indicate interactions with photosynthetic organisms.

The phylum Bacteroidetes was significantly more abundant when incubated with EPS_HB_ and with EPS_coll_. Unlike the Verrucomicrobia, the Bacteroidetes are well known for their capacity to degrade biopolymers, including EPS (Haynes et al., 2007; McKew et al., 2013). Many OTUs from diverse classes of Bacteroidetes were selectively enriched (Figures 5B, 6B), two of which were enriched under both EPS additions, but had a higher relative abundance with EPS_HB_. One of these, from the genus *Tenacibaculum*, constituted 12% of the community with EPS_coll_ and 20% with EPS_HB_, and has been shown to possess enzymes able to degrade a great range of organic compounds from a wide range of marine habitats including tidal flat sediments (Suzuki et al., 2001; Frette et al., 2004; Choi et al., 2006; Jung et al., 2006). The second Bacteroidetes OTU, 2-fold more abundant in EPS_HB_ than EPS_coll_, was from the genus *Algoriphagus*, which is able to degrade an array of different compounds as carbon and energy sources (e.g., D-glucose, D-galactose, sucrose) (Yoon et al., 2005; Alegado et al., 2013). This genus is normally found in diverse marine habitats, such as seawater, tidal mudflats, and salterns, and also associated with algae (Nedashkovskaya et al., 2004) or cyanobacterial mats (Bowman et al., 2003). Such features indicate that *Algoriphagus* species might be considered as specialist EPS/carbohydrate degraders.

Similarly to Verrucomicrobia, certain taxa within the phylum Planctomycetes are emerging as important biopolymer-degrading microbes (Wang et al., 2015; Yilmaz et al., 2016). They generally are enriched in marine snow (Fuchsman et al., 2012), are associated with macroalgae (Lage and Bondoso, 2011), and increase in abundance in coastal diatom blooms (Morris et al., 2006), suggesting that they may be able to utilize the released algal organic carbon. Here, Planctomycetes (class Phycisphaerae) was more abundant when grown on EPS_coll_ compared to NoAdd-EPS, with three OTUs specifically enriched (Figure 5), but not when incubated with EPS_HB_ compared to NoAdd-EPS (Figure 6). One of the most EPS_coll_-enriched OTUs was from the genus *Rhodopirellula*. *Rhodopirellula baltica* degrades carbohydrates in marine environments (Gade et al., 2005) and genome sequencing revealed its ability to degrade algal-derived sulfated polysaccharides (Glöckner et al., 2003; Wegner et al., 2013). However, in contrast to the current finding, previous studies performed in the Colne estuary using different approaches did not detect this Planctomycetes as a specialist EPS degrader (Hofmann et al., 2009; McKew et al., 2013; Taylor et al., 2013), thus further work is needed to clarify the role of this phylum in intertidal systems.

The relative similarity of many taxa in the +EPS_coll_ and +EPS_HB_ treatments could indicate that there is a general capability of utilizing EPS, in accordance with previous studies. Miyatake et al. (2014), for example, showed that of the bacterial taxa targeted all incorporated diatom-derived material. For the Colne, previous studies showed only small changes in overall bacterial community composition in response to added EPS using different approaches (Hanlon et al., 2006; Haynes et al., 2007; Bellinger et al., 2009). Taylor et al. (2013) demonstrated that a diverse range of bacterial taxa were enriched when exposed to cEPS, including highly enriched Alphaproteobacteria and Gammaproteobacteria, but of different lower-order taxa to those found here. Although our results cannot preclude uptake of EPS by a wide range of bacteria, which express this capacity depending on the particular environmental conditions or composition of EPS, the results of the present experiment show clear and consistent changes in the abundance of several taxa when incubated with added EPS. This is consistent with other studies (Hanlon et al., 2006; Haynes et al., 2007; Taylor et al., 2013) and demonstrates some specialization for degradation of different types of EPS (EPS_coll_ and EPS_HB_).

## Conclusions

EPS constitute a large fraction of the available carbon and energy in marine sediments and wherever phototrophic microbes abound (Underwood et al., 2005; Bellinger et al., 2009). Here, we have shown that fractions of EPS with different structural complexity (operationally termed colloidal and hot-bicarbonate extracted) were degraded, at higher rates compared with those reported previously (Table 2), contributing to the transfer of organic C from microphytobenthos to heterotrophic bacteria. The comparison of degradation rate constants for the different organic fractions studied here, using a one-G exponential decay model, confirmed that DOC and cEPS fractions from the +EPS_HB_ treatment are more refractory than their counterpart fraction in the +EPS_coll_ treatment (Table 1) in accordance with most studies, where higher molecular weight, complex compounds have a lower degradability (Table 2). In addition, our results indicate that EPS, particularly the EPS_HB_ fraction, contain large amounts of N and P that may be released during EPS degradation at rates high enough to support microbial growth in slurries. The relevance of the EPS_HB_ fraction as a source of regenerated nutrient, mainly N and P, for the sediment microbial community *in situ* and the observed differences with respect to EPS_coll_ fraction require further investigation. The addition of different diatom-derived EPS also induced the enrichment of different bacterial taxa, indicating the existence of some specialization for degradation of different types of EPS. Given the widespread use of high-throughput amplicon sequencing, programs are being developed to infer microbial functions based on phylogeny. However, such approaches must be grounded on solid experimental evidence as presented here, considering the complex interactions of EPS degradation in sediments (Bellinger et al., 2009; McKew et al., 2013; Taylor et al., 2013). Further investigation is required to understand how changes in nutrient regeneration and EPS degradation rates and the differential enrichment of distinct taxa affect EPS budgets in intertidal sediments *in situ*, in relation to changes in the relative composition of EPS during a tidal or seasonal cycle.

## Author contributions

All the authors designed the experiment. JB carried out the experiment and analyzed the samples. All authors analyzed and interpreted the data and wrote the manuscript.

## Funding

The research was funded by Projects CTM-2009-10736, CTM2013-43857-R (Ministry of Economy and Competitiveness, Spain), and P11-RNM-7199 (Andalusian Regional Government). JB was funded by a FPI Grant (BES-2010-035711) from the Ministry of Economy and Competitiveness, Spain. GU and TM were funded by a NERC grant NE/K001914/1 (Data Synthesis and Management of Marine and Coastal Carbon).

### Conflict of interest statement

The authors declare that the research was conducted in the absence of any commercial or financial relationships that could be construed as a potential conflict of interest.
