# Supplementary material for: Different Types of Diatom-Derived Extracellular Polymeric Substances Drive Changes in Heterotrophic Bacterial Communities from Intertidal Sediments
Source: Front Microbiol. 2017 Feb 27;8:245. doi: 10.3389/fmicb.2017.00245 (PMC5326797; doi:10.3389/fmicb.2017.00245)
Supplement: Supplementary file 1 [file DataSheet1.docx]

Supplementary Material

Different types of diatom-derived extracellular polymeric substances drive changes in heterotrophic bacterial communities from intertidal sediments

Julio Bohórquez^1, 2, *^, Terry J. McGenity^2^, Sokratis Papaspyrou^3^, Emilio García-Robledo^1, 4^, Alfonso Corzo^1^ and Graham J. C. Underwood^2^

^1^ Department of Biology, Faculty of Marine and Environmental Science, University of Cádiz, Pol. Río San Pedro s/n. 11510 Puerto Real, Spain.

^2^ School of Biological Sciences, University of Essex, Wivenhoe Park, Colchester, Essex CO4 3SQ, UK.

^3^ Departamento de Biomedicina, Biotecnología y Salud Publica, Universidad de Cádiz, Polígono Rio San Pedro s/n, 11510 Puerto Real, Spain.

^4^ Microbiology Section, Department of Biosciences. University of Aarhus. Ny Munkegade 116, DK-8000, Aarhus, Denmark.

*** Correspondence:** Julio Bohórquez: [julio.bohorquez@uca.es](mailto:julio.bohorquez@uca.es)


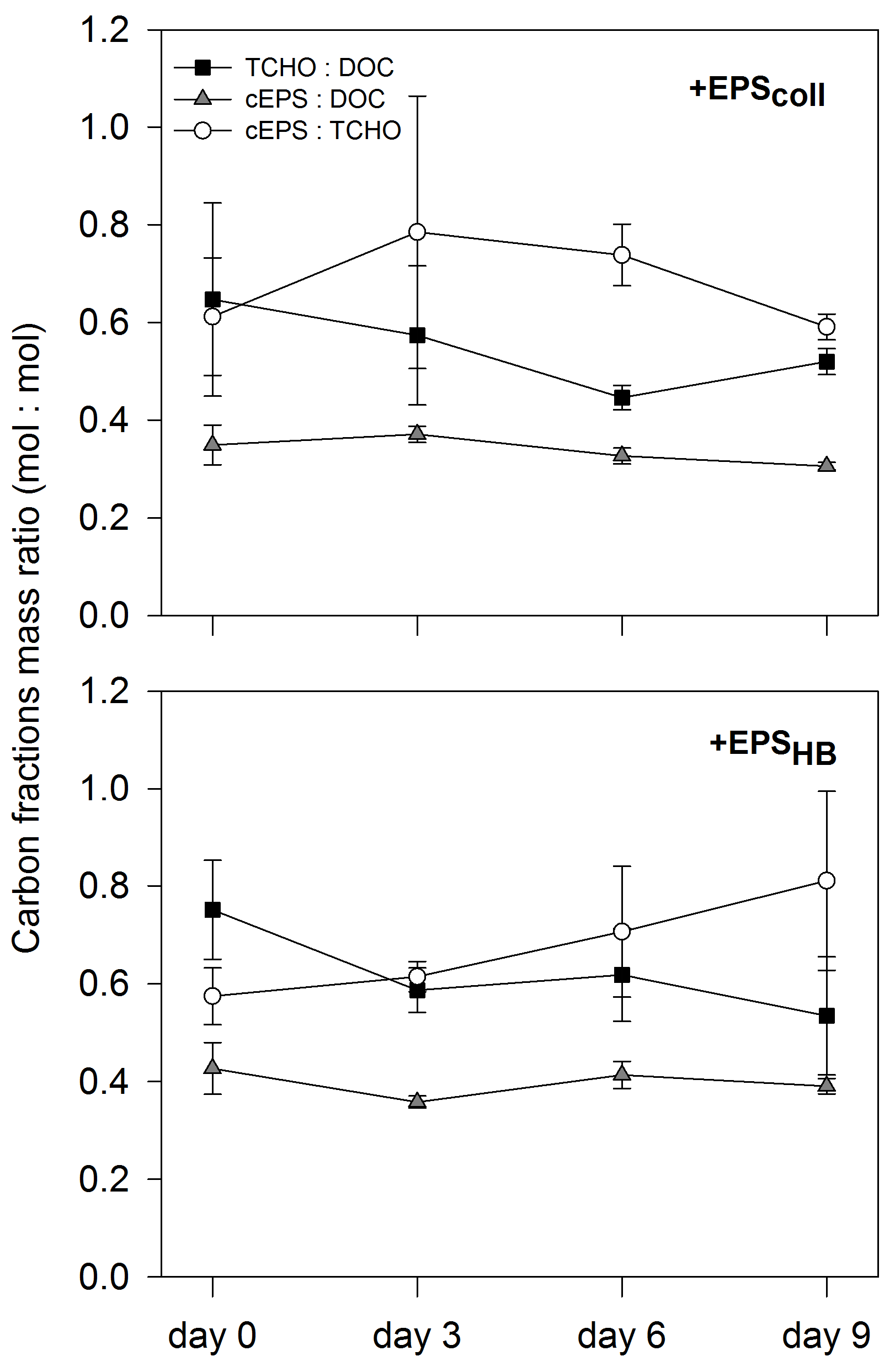


**Supplementary Figure 1. Changes in TCHO : DOC, cEPS : DOC and cEPS : TCHO ratios (mol : mol) in estuarine sediment slurries (200 mg from the top 2 mm, 80 ml artificial sea water (ASW) over 9 days) for the treatments +EPS_coll_, and +EPS_HB_.** Values are means (n=3) ± standard error (SE).


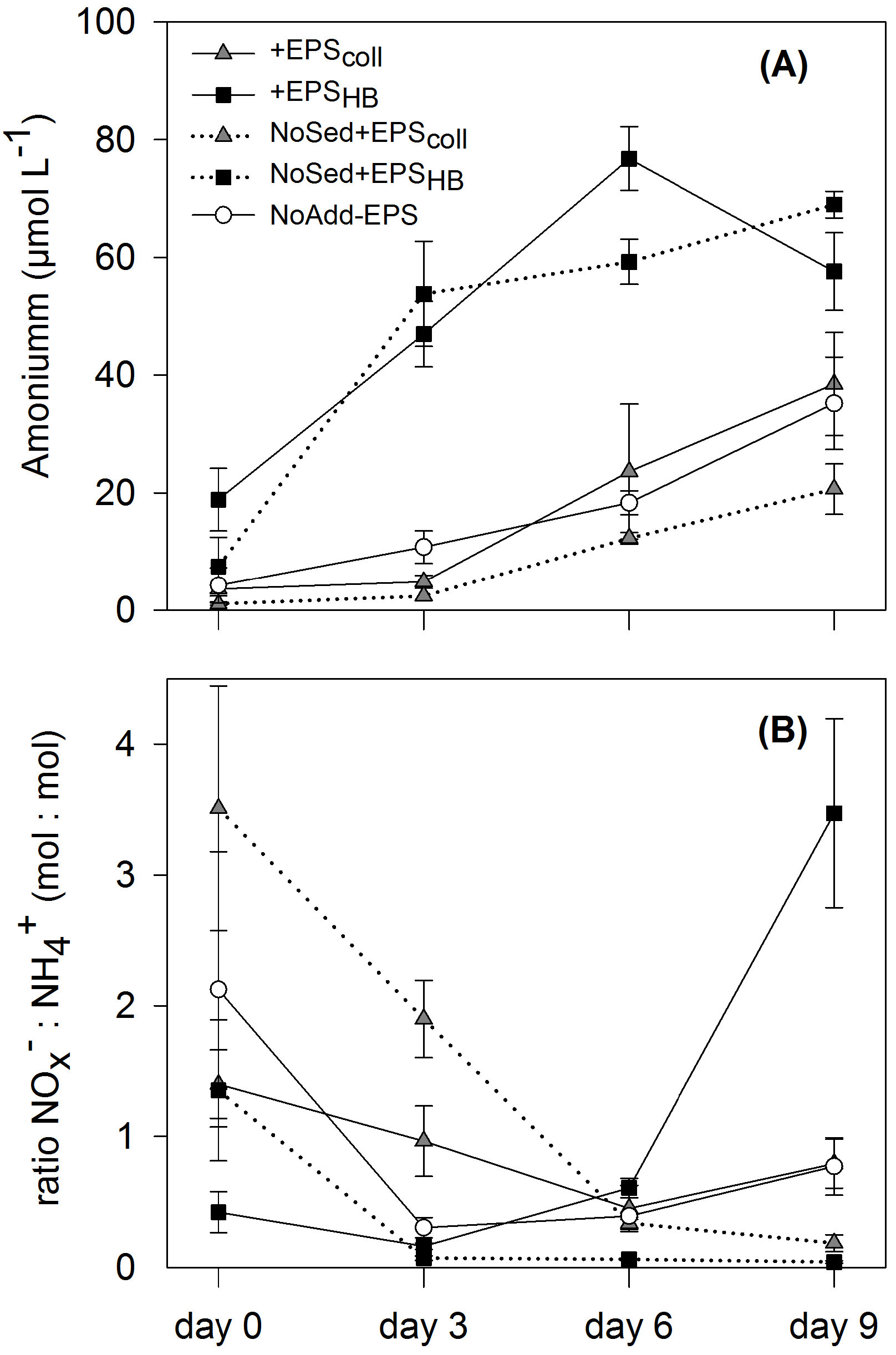


**Supplementary Figure S2.** **Concentrations of Ammonium (µmol L^-1^ of slurry) (A) and the nitrogen oxides (NO_2_^-^ + NO_3_^-^) : ammonium ratio (mol : mol) (B) in estuarine sediment slurries (200 mg from the top 2 mm, 80 ml artificial sea water (ASW) over 9 days) for the treatments +EPS_coll_, +EPS_HB_, NoAdd-EPS and the controls (not sediment inoculum added) NoSed+EPS_coll_ and NoSed+EPS_HB_ represented with dotted lines.** Values are means (n=3) ± standard error (SE).

**
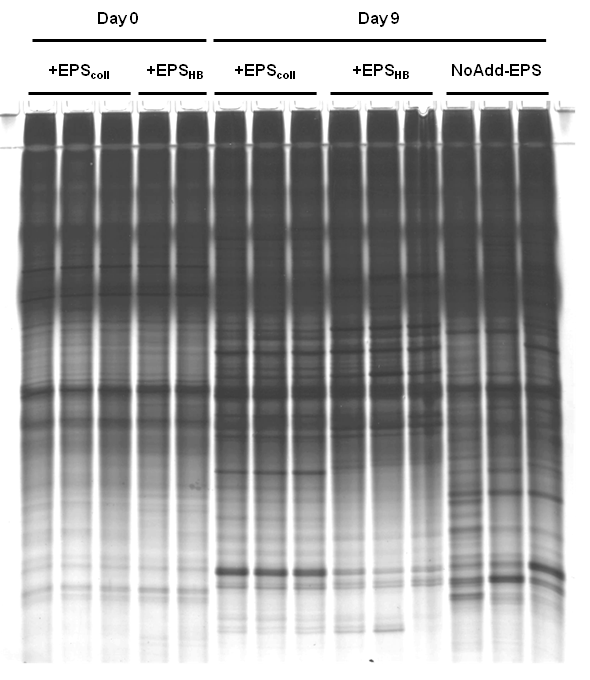
**

**Supplementary Figure S3. Bacterial community DGGE gel profile for DNA extracted from sediment slurries at day 0 and day 9 for the treatments +EPS_coll_, +EPS_HB_ and NoAdd-EPS.** DGGE Bands of treatments +EPS_coll_ and +EPS_HB_ did not show differences at day 0 and were used as initial samples for the three treatments +EPS_coll_, +EPS_HB_ and NoAdd-EPS. The sampling days (day 0 and day 9) are indicated above the image for the corresponding treatment. Replicates of samples are shown.


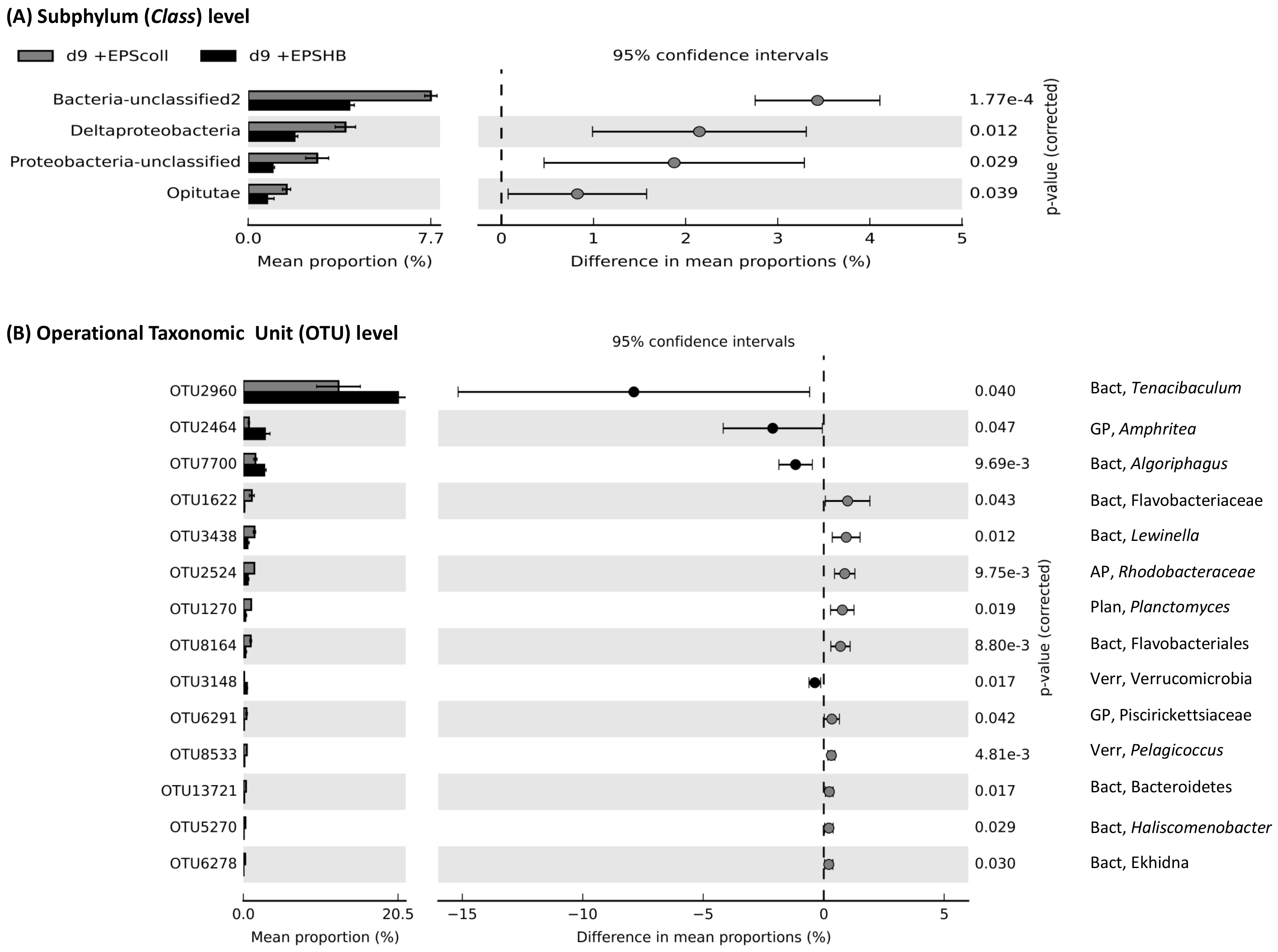


**Supplementary Figure S4. Comparison of bacterial community profiles in +EPS_coll_ versus +EPS_HB_ by day 9 both at the levels of subphylum (class) (A) and at Operational Taxonomic Unit (OTU) defined at 95% similarity (B).** Analysis was performed using STAMP (Parks *et al*. 2010, 2014) with default parameters except that parameters for filtering out were: p value >0.05; difference between proportions <0.2 or difference between ratios <1.5. Data were sorted according to effect size. Note the differences in the scale of the x axes. No identified phyla were differentially abundant between the two treatments with different classes of EPS (not shown). The information to the right of the p values is the identity of the OTU, whereby the phylum is indicated to the left of the comma (AP = Alphaprotebacteria; Bact = Bacteroidetes; GA = Gammaproteobacteria; Plan = Planctomycetes; Verr = Verrucomicrobia), and the lowest taxonomic level to which the OTU can be confidently assigned is indicated to the right of the comma. A total of 10 bacterial taxa were significantly enriched in +EPS_coll_ treatment compared to 4 that significantly increased in +EPS_HB_.

**Supplementary Table S1. Samples from the microcosms subjected to pyrosequence analysis of bacterial 16S rRNA genes, and associated information to allow data to be extracted from the European Nucleotide Archive (ENA) under accession number PRJEB15429.**

| **Sample name** | **Treatment name** | **Type of EPS extract added** | **Time of sampling (days)** | **Lane ^1^** | | **Mid ^1^** | | **Mid sequence ^1^** |  | | |
| --- | --- | --- | --- | --- | --- | --- | --- | --- | --- | --- | --- |
| d0 +EPS_coll_ | +EPS_coll_ | colloidal EPS | 0 | 2 | 175.MID.76 | | ACATGACGAC | | |  |  |
| d0 +EPS_coll_ | +EPS_coll_ | colloidal EPS | 0 | 2 | 176.MID.77 | | ACGACAGCTC | | |  |  |
| d0 +EPS_coll_ | +EPS_coll_ | colloidal EPS | 0 | 2 | 177.MID.78 | | ACGTCTCATC | | |  |  |
| d9 +EPS_coll_ | +EPS_coll_ | colloidal EPS | 9 | 2 | 178.MID.79 | | ACTCATCTAC | | |  |  |
| d9 +EPS_coll_ | +EPS_coll_ | colloidal EPS | 9 | 2 | 179.MID.80 | | ACTCGCGCAC | | |  |  |
| d9 +EPS_coll_ | +EPS_coll_ | colloidal EPS | 9 | 2 | 180.MID.81 | | AGAGCGTCAC | | |  |  |
| d9 +EPS_HB_ | +EPS_HB_ | Hot-bicarbonate EPS | 9 | 2 | 181.MID.82 | | AGCGACTAGC | | |  |  |
| d9 +EPS_HB_ | +EPS_HB_ | Hot-bicarbonate EPS | 9 | 2 | 182.MID.83 | | AGTAGTGATC | | |  |  |
| d9 +EPS_HB_ | +EPS_HB_ | Hot-bicarbonate EPS | 9 | 2 | 183.MID.84 | | AGTGACACAC | | |  |  |
| d9 NoAdd-EPS | NoAdd-EPS | none | 9 | 2 | 184.MID.85 | | AGTGTATGTC | | |  |  |
| d9 NoAdd-EPS | NoAdd-EPS | none | 9 | 2 | 185.MID.86 | | ATAGATAGAC | | |  |  |
| d9 NoAdd-EPS | NoAdd-EPS | none | 9 | 2 | 186.MID.87 | | ATATAGTCGC | | |  |  |

^1^ From accession PRJEB15429, the specific samples may be identified by clicking the “Runs” tab, and using the unique combination of elements within the file name: Lane number and Mid number. The sequence of the Mid (10-base extended multiplex identifier) is provided for information, but, along with other primer sequence, has been removed from deposited sequences.

Nucleotide sequences of the fusion primers are as follows:

*Forward primer* 5´‐CGTATCGCCTCCCTCGCGCCATCAG‐(TAGACTGCAC)‐(Bact-341F)‐3´

*Reverse primer* 5´‐CTATGCGCCTTGCCAGCCCGCTCAG‐(MID1-99)‐(Bact-805R)‐3´
